# Etiologic Profile of the Pneumococcus in Ghana: A Systematic Review

**DOI:** 10.1155/2024/8368996

**Published:** 2024-08-27

**Authors:** Reuben E. Arhin, Eric S. Donkor, Hans-Christian Slotved, Fleischer C. N. Kotey, Nicholas T. K. D. Dayie

**Affiliations:** ^1^ Department of Medical Microbiology University of Ghana Medical School, Korle Bu, Ghana; ^2^ Department of Science Laboratory Technology Accra Technical University, Accra, Ghana; ^3^ Department of Bacteria Parasites and Fungi Statens Serum Institute, Copenhagen, Denmark

**Keywords:** Carriage, Etiologic profile, Ghana, Meningitis, PCV-13, Pneumococci, *Streptococcus pneumoniae*

## Abstract

**Objective:** To describe the profile of *Streptococcus pneumoniae*, identify research gaps, and provide in-depth insights into various aspects related to the pathogen.

**Methods:** Google Scholar, PubMed, and ScienceDirect were searched for all studies on the pneumococcus in Ghana that reported on specimen collected, population and sample size, carriage prevalence, incidence of pneumococcal diseases, age of the study population, types of test performed, serotypes identified, antimicrobial susceptibilities, or molecular analysis on the pneumococci for data extraction.

**Results:** Overall, a total of 7954 results were obtained from the three-database search, and of this, 24 articles were selected after screening. A total of 924 isolates were accounted for by serotyping/serogrouping. The prevalence of pneumococcal carriage in Ghana ranges from 11.0% to 51.4% in the population depending on the age (≤ 24–80 years), sickle cell disease (SCD), human immunodeficiency virus (HIV) status, or health of the study population, and penicillin (Pen)-nonsusceptible isolates ranged from 17% to 63%. The prevalence of pneumococci found as the etiologic agent of diseases among Ghanaians ranges from 3.4% for otitis media to 77.7% for meningitis. Overall, the 13-valent pneumococcal conjugate vaccine (PCV) (PCV-13) carriage serotypes accounted for 28.4% of the reported pneumococcal isolates. PCV-13 invasive serotypes accounted for 22.4% of the reported isolates. The non-PCV-13 carriage serotypes accounted for most (43.9%) of the reported isolates. In the pre-PCV-13 era, the nontypeable (NT) (5.5%) and other nonvaccine types (NVTs) (6.4%) were reported as being predominant. The non-PCV-13 serotypes accounted for 4.4% of the reported isolates in invasive pneumococcal disease (IPD) cases. Multidrug resistance (MDR) ranged from 7.8% to 100%.

**Conclusion:** Predicting the invasiveness of pneumococci using molecular typing is the way to go in the future as this will provide answers to the extent to which capsular switching is contributing to the pneumococcal disease burden in Ghana almost a decade after introducing PCV-13. Continuous monitoring of antibiotic resistance patterns at both phenotypic and genotypic levels, along with serotyping and molecular typing, should be a standard practice in the surveillance of pneumococcal disease burden in Ghana.

## 1. Introduction

*Streptococcus pneumoniae*, also known as pneumococci, have received a vast global attention due to their pathogenicity [1], antibiotic resistance [2], and disease burden [3, 4] in human populations. In Ghana, pneumococci are associated with invasive diseases including frequent outbreaks of meningitis, especially in the northern part of the country that falls in what has been termed as the “meningitis belt” stretching from West to East Africa [5]. Due to this, Renner et al. have emphasized on the importance of continuous monitoring of the pneumococci particularly in Africa [6]. Thus, pneumococci have received extensive attention in Ghana leading to the introduction of childhood vaccination with the 13-valent pneumococcal conjugate vaccine (PCV-13) in the year 2012 [5]. This was expected to reduce the burden of invasive pneumococcal diseases (IPDs) in children as well as the prevalence of drug-resistant pneumococci [7]. Despite these efforts in combating pneumococcal diseases, the introduction of PCV-13 into the childhood immunization scheme in Ghana has not been entirely successful. This is partly because the vaccine does not wholly incorporate all the serotypes that are found in reported cases of IPDs in Ghana, coupled with the problem of capsular switching [8]. Furthermore, PCV-13 serotype meningitis in children < 5 years decreased only by 51% in the post-PCV era (2013–2016) [6], and 33.1% of 1006 meningitis cases reported in the northern regions of Ghana between December 2015 and April 2016 were due to pneumococci [5]. These necessitate surveillance of the pneumococcus in Ghana to prevent the high disease burden associated with the pathogen.

This study is aimed at performing a literature review on pneumococci in Ghana. The primary objective was to describe the profile of *Streptococcus pneumoniae*, identify research gaps, and provide in-depth insights into various aspects related to the pathogen. These aspects included the distribution of carriage- and invasive-related serotypes, the impact of PCV-13 vaccination, disease burden, antimicrobial resistance, and clonality of the pathogen.

## 2. Materials and Methods

The guidelines for the Preferred Reporting Items for Systematic Reviews and Meta-Analyses were followed in this systematic review [9]. Data extraction was done by three authors to reduce bias, using the search terms “*Streptococcus pneumoniae*” or “Pneumococci” or “Pneumococcus” or “Pneumococcal” in combination with “Ghana” or “Ghanaian” on Google Scholar, PubMed, and ScienceDirect.

On Google Scholar, the search terms “*Streptococcus pneumoniae* OR pneumococci OR pneumococcus AND Ghana OR Ghanaian” were used to search all fields for related articles from 2000 to August 2023.

On PubMed, filters were applied to look for “abstracts”, “free full text”, “full text of clinical trials”, “observational studies”, and “randomized control trials” in “English” by searching for the terms “*Streptococcus pneumoniae*” or “Pneumococci” or “Pneumococcus” or “Pneumococcal” in combination with the search terms “Ghana” or “Ghanaian” in all fields from 2000 to August 2023. Individual searches using the titles were also done.

On ScienceDirect, the advanced search item “(“Streptococcus pneumoniae” OR pneumococci OR pneumococcus) AND (Ghana OR Ghanaian)” was used to find all related articles from 2000 to August 2023. Filters were applied to look for research articles.

**Figure 1:**
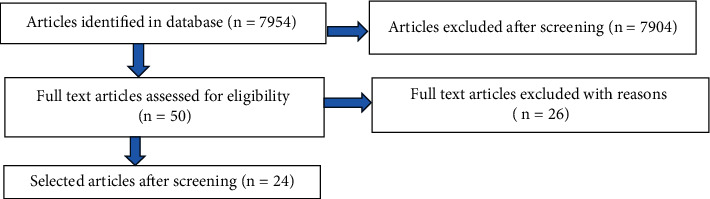
Flow diagram indicating the process in eliminating unrelated articles.

The titles were screened in relation to the topic, and the abstracts were studied before proceeding to the main content. In addition, the quality of each study was assessed by checking the rationale, objectives, study design, setting, measured variables, and key results. All related studies that reported on at least four of the following were included for data extraction: (1) prevalence of isolates, (2) sample size, (3) age of study subjects, (4) study setting, (5) period of study, (6) type of morbidity or carriage, (7) serotypes of pneumococci, (8) antimicrobial susceptibility test (AST), and (9) molecular analysis of pneumococci isolates from Ghana were included. Of these, 24 studies were identified as being relevant or related to works on pneumococci conducted in Ghana and hence were included (Figure 1). Studies that did not involve data from Ghana were excluded. All other related studies from Ghana that did not meet the criteria for data extraction were only cited in the discussions.

## 3. Results

Of 7650 search results from Google Scholar, 24 were selected for data extraction. Of this, 21 were not relevant to the topic, and the rest were not related to Ghana. Of 110 search results from PubMed, none were selected since these were not relevant to the topic. However, an individual search of the titles yielded a repeat of the articles selected from Google Scholar on the PubMed database. Of 194 search results from ScienceDirect, 181 were not from Ghana, and five were not relevant to the topic. Overall, a total of 7954 results were obtained from the three-database search, and of this, 24 articles were selected after screening (Table 1). A total of 924 isolates were accounted for by serotyping/serogrouping.

The prevalence of pneumococcal carriage in Ghana ranged from 11% to 51.4% in the population depending on the age (≤ 24–80 years), sickle cell disease (SCD), human immunodeficiency virus (HIV) status, or health of the study population. The highest carriage prevalence was reported for healthy children under 5 years of age, and the lowest prevalence was for HIV patients of ages up to 80 years. The carriage studies have mainly focused on hospital settings and schools (Table 2). The prevalence of Pen-nonsusceptible isolates ranged from 17% to 63% (Table 2). The highest prevalence was for healthy children under 6 years of age. Pen-nonsusceptible isolates include both isolates identified as Pen-resistant and those that were found to be nonsusceptible during screening.

Pneumococci have been the predominant pathogen isolated from invasive diseases in some cases in Ghana. They have been found mostly to be the cause of meningitis. The prevalence of pneumococci found as the etiologic agent of diseases among Ghanaians ranges from 3.4% for otitis media to 77.7% for meningitis.

Isolates for the studies showing the distribution of PCV-13 carriage in Ghana (Table 3) were obtained from children in nursery and kindergarten schools in Accra and Tamale metropolis [7], children under 5 years of age at a paediatric healthcare center [25], children with HIV at Korle Bu Teaching Hospital in Accra [19], patients with SCD at the Korle Bu Teaching Hospital and Princess Marie Louis Children's Hospital [8], HIV patients under 5 years of age up to 80 years old [12], and children in kindergarten and immunization centers in Cape Coast [26].

Overall, the PCV-13 carriage serotypes accounted for 28.4% of the reported pneumococcal isolates (Table 3). The predominant ones were serotypes 19F (6.4%), 23F (4.6%), 6A (3.5%), 9V (3.0%), 6B (2.9%), 14 (2.5%), 3 (2.4%), and 19A (1.3%). Serotypes 18C (1.3%), 7C (0.4%), 4 (0.3%), and 1 (0.1%) were the least reported among the PCV-13 carriage isolates.

Also, serotypes 19F (4.0%), 9V (2.6%), 23F (2.3%), and 3 and 14 (1.1% each) were the predominant PCV-13 carriage serotypes prior to the introduction of PCV-13 into Ghana's Expanded Programme on Immunization [7]. Serotypes 1, 6B, and 7C were not reported in carriage studies in the pre-PCV-13 era [7]. Relatively fewer PCV-13 carriage serotypes (3, 4, 6A, 9V, 14, 18C, 19A, 19F, and 23F) were reported in the pre-PCV-13 era than in the PCV-13 era (1, 3, 6A, 6B, 9V, 14, 18C, 19A, 19F, and 23F).

Overall, the PCV-13 invasive serotypes accounted for 22.4% of the reported isolates (Table 4). Serotypes 1 (20%) and 3 (1%) were the predominant cause of IPDs. Serotypes 5 (0.4%), 23F (0.3%) and 14 and 4 (0.2% each) were also identified in cases of IPDs. Serotypes 6A, 19A, and 19F (0.1%) were the least reported serotypes in cases of IPDs among the PCV-13 serotypes.

Prior to the introduction of PCV-13 in Ghana's Expanded Programme on Immunization, serotypes 1 (6.8%) and 3 (0.8%) were also the predominant cause of PCV-13 serotype IPDs [23]. Relatively fewer PCV-13 invasive serotypes (1, 3, 4, 6A, and 14) were reported in the pre-PCV-13 era than in the post-PCV-13 era (1, 3, 4, 5, 14, 19A, 19F, and 23F).

Isolates for the studies showing the distribution of non-PCV-13 carriage serotypes in Ghana (Table 5) were obtained from children in nursery and kindergarten schools in Accra and Tamale metropolis [7], children under 5 years of age at a paediatric healthcare center [25], children with HIV at Korle Bu Teaching Hospital in Accra [19], patients with SCD at the Korle Bu Teaching Hospital and Princess Marie Louis Children's Hospital [8], HIV patients under 5 years of age up to 80 years old [15], and children in kindergarten and immunization centers in Cape Coast [26].

Overall, the non-PCV-13 carriage serotypes accounted for most (43.9%) of the reported isolates (Table 5). Of this, the nontypeable (NT) isolates were predominant (7.2%). Of the typeable isolates, the predominant reported ones were serotype 23B (3.6%), 11A (2.8%), Serogroup 19 (2.4%), serotype 15B (2.1%), serotype 13 (1.5%), serotype 34 and 10A (1.4% each), 16F (1.3%), 8 (1.2%), 15A (1%), and 32F (0.7%). Serotypes 33F, 35B, and 38 (0.2% each) were few. Serotype 33A, Serogroup 33, 24F, 23A, 21/29, 19/14, 6/19, 18B, 17A, 11D, 9A, 9, and 6/10 were the least reported (0.1% each).

In the pre-PCV-13 era, the NT (5.5%) and other nonvaccine types (NVTs) (6.4%) were reported as being predominant (Table 5). Of the typed non-PCV-13 carriage isolates, serotype 11A (1.1%) and serotype 8 and 15B (0.8% each) were predominant during the pre-PCV-13 era [7].

Overall, the non-PCV-13 serotypes accounted for 4.4% of the reported isolates in IPD cases (Table 6). Of this, most of the cases were due to serogroups 12/44/46 (2.1%), serotypes 12F (0.9%), and 8 (0.4%). Some of the cases were due to serogroups 6 and 18 and serotypes 7F and 35B (0.2% each). Few of the cases were due to serotypes 10F and 38 (0.1% each).

However, in the pre-PCV-13 era, serotypes 8 (0.4%) and 12F (0.2%) accounted for most of the IPDs caused by the non-PCV-13 serotypes [23]. Relatively, fewer non-PCV-13 invasive serotypes (7F, 8, 10F, 12F, and 38) were reported in the pre-PCV-13 era than in the PCV-13 era (6, 7F, 12F, 12/44/46, 18, and 35B).

The antimicrobial resistance of pneumococcal isolates from Ghana (Table 7), obtained from blood, CSF, and nasopharyngeal swabs, has been determined using the following antibiotics: penicillin (Pen), erythromycin (Ery), levofloxacin (Lev), tetracycline (Tet), gentamicin (Gen), amoxicillin (Amo), ampicillin (Amp), trimethoprim (Tmp), sulfamethoxazole (Smz), chloramphenicol (Chl), ciprofloxacin (Cip), cloxacillin (Clo), cefuroxime (Crx), ceftriaxone (Ctr), oxacillin (Oxa), and cefotaxime (Ctx). Multidrug resistance (MDR) from these studies ranges from 7.8% to 100% depending on the status of the study population which included healthy carriers, subjects with morbidities, and at-risk populations such as those with SCD condition and people living with HIV infection. Resistance to Amp, Clo, vancomycin, rifampicin, and lincomycin (0% each) was not reported. Antimicrobial resistance was low for Lev (0%–2.5%), clindamycin (0%–5.3%), Ctr (0%–5.6%), Crx (11%), Gen (16.7%–25%), and Oxa (27.8%–37.3%), whereas it was high for Cip (3.4%–57%), Ery (0%–66.7%), Chl (0%–75%), Pen (0%–76%), Amo/Amp (77%), Tet (26%–83.3%), Ctx (0%–100%), cotrimoxazole (76%–100%), and Ery/azithromycin (100%).

## 4. Discussion

### 4.1. Pneumococcal Carriage and Dominant Serotypes

Although it is known that carriage and serotype prevalence have been influenced by the introduction of PCV-13 in 2012, it is not clear to which extent. In a systematic review and meta-analysis, including data from Ghana, pneumococci carriage was reported to be frequent among children under 5 years [32]. A study to examine the relationship between household air pollution and infant microbial nasal carriage in Ghana identified pneumococci as the third highest predominant bacteria in carriage after *Haemophilus influenza* and *Moraxella catarrhalis* [33]. Dayie et al. reported an average carriage of 32 % in Accra Metropolis and Tamale Metropolis before the introduction of PCV-13 into Ghana's Expanded Programme on Immunization [7]. After the introduction of PCV-13, Dayie et al., in a study on pneumococcal carriage among SCD patients at the Korle Bu Teaching Hospital and Princess Marie Louis Hospital, reported a carriage prevalence of 39.1% for SCD children and 10% for SCD adults [8]. Among HIV/AIDS patients studied in these same facilities, the prevalence was 25% and 7.3% for children and adults, respectively [15]. A similar study by Donkor et al. reported a carriage prevalence of 30.5% among children with HIV at the KBTH [19] (Table 2). Variations in the prevalence among groups of different health status suggest that carriage may vary depending on the biological state of the groups under consideration. For example, carriage among HIV subjects, diabetics, and sickle cell subjects may vary from the healthy population due to factors such as intake of medication [12, 13] by these at-risk groups, and this may contribute to differences between the carriage prevalence between these groups. Compared to other countries in West Africa such as Nigeria, the prevalence of carriage in Ghana appears to be relatively low. Adetifa et al. reported a post-PCV10-era pneumococcal carriage prevalences of 73.7% and 50.1% in a two-site population-based survey of Nigeria [34] and 85.4% in infants in post-PCV-13 Gambia [35]. In a descriptive multicenter study covering five countries, namely, Cambodia, Mongolia, China, India, Madagascar, Mali, Haiti, and Paraguay carriage prevalences in children aged 0–60 months have been reported as 68.2% for pneumoniae cases and 47.5% for controls in clinical settings [36]. These are still higher than what have been reported in clinical settings in Ghana. Also, an overall carriage prevalence of 48% was reported in children and adults in Denmark following the introduction of PHiD-CV10 in 2011 [37] which is also high compared to the Ghanaian example.

### 4.2. Impact of PCV-13 Introduction

However, it has been previously reported that PCV-13 has neither reduced the vaccine serotypes in the receiving population, nor has it reduced carriage [6]. Aside from this, vaccination of infants with PCV-13 is expected to produce a herd effect against pneumococcal diseases as observed in high-income countries practicing the 2 + 1 schedule, but the scale of vaccination required to achieve this indirect impact in African countries like Ghana is unknown [38]. In Ghana, since 2013, the current coverage going by the 3 + 0 schedule has exceeded 88%, but meningitis outbreaks are recurrent events [39]. Abbey et al. identified that PCV-13 has been used in Ghana since 2012, but the vaccine offers protection for only 13 serotypes responsible for IPDs among the other serotypes that are prevalent in Ghana [40]. It was reported that although PCV-13 had reduced the number of IPD cases, there remains a significant disease burden because of the nonvaccine serotypes [40]. A descriptive review of secondary data on reported cases of meningitis from January 2010 to December 2015 by Kaburi et al. showed that in the northern regions of Ghana, pneumococcal meningitis was still endemic despite the availability of pneumococcal vaccines since 2012 [41]. It has therefore been proposed that a reactive vaccine approach, like what is in place for meningococcal meningitis outbreaks, should be deployed during pneumococcal epidemics to reduce the mortality [38]. Hence, there is the need for surveillance to continue to monitor the possible benefit such an approach would produce since PCV-13 has a limited coverage of the serotypes.

Of the more than 100 known serotypes of pneumococci [42], studies in Ghana have identified about 44 different serotypes in carriage and disease cases (Tables 3, 4, 5, and 6). This again stands relatively low compared to the 65 different serotypes that have been previously reported by Adetifa et al. [34]. Some of these serotypes reported in Ghana have been implicated in meningitis outbreaks alongside the notorious Serotype 1. Carriage studies to investigate the prevalence of the vaccine serotypes after a decade of PCV-13 in Ghana will be necessary to further evaluate the impact of the vaccine not just on the vaccinated population but also its herd effect on the older unvaccinated population.

### 4.3. Prevalence of Pneumococcal Diseases

Pneumococcal diseases include both invasive and noninvasive diseases [43]. Both contribute to the pneumococcal disease burden in Ghana. According to Renner et al., it is essential to maintain continuous monitoring of pneumococci, especially in Africa. This is primarily due to the high disease burden in the region, the fact that bacterial carriage serves as a precursor to the disease, and the persistence of vaccine serotypes in the post-PCV13 era in Ghana, despite their replacement by nonvaccine serotypes [6].

Cerebrospinal meningitis is known to be primarily caused by pneumococci among its major etiological agents [5, 42]. In Ghana, the pneumococcus is the main course of bacterial meningitis, and this poses a public health crisis especially in the northern parts of the country which fall within the meningitis belt [44]. The disease burden due to pneumococcal meningitis is greater than that of meningococcal meningitis [38]. Bozio et al. identified pneumococci as being the cause of meningitis in 153 of 409 CSF samples and reported an observed incidence rate of 4.33 for 100,000 population within a period of 14 weeks from 2016 to 2017 [12]. In a retrospective study conducted at the Komfo Anokye Teaching Hospital, pneumococci were found to be the highest prevalent cause of bacterial meningitis (77.7%) [31]. Similarly, a study in Jaman North reported that 77.3% of recorded cases of meningitis outbreak in the district from a retrospective study were due to pneumococci [13]. Pneumococci were also found to be the most prevalent bacterial cause (50 of 73 [68.5%]) of meningitis in a global surveillance for vaccine-preventable invasive bacterial diseases targeting Ghana [6]. Nuoh et al. reported on meningitis surveillance data from the Upper West Region and identified that 48.2% of bacterial meningitis was caused by pneumococci [45].

Aku et al. have reported that high coverage of the vaccine may have accounted for a reduction in pneumococcal infection rates among the younger age group [5]. Vaccine immunity is known to be serotype-specific and, as such, is expected to offer protection mainly against the PCV-13-included serotypes and hence possibly leave the population vulnerable to the non-PCV-13 serotypes. Abbey et al. reported that there is limited data on the extent to which the nonvaccine serotypes cause IPDs in Ghana [40]. Another question that remains to be answered is the extent to which the vaccine and nonvaccine serotypes are contributing to the pneumococcal disease burden in Ghana almost a decade after introducing PCV-13. In Senegal, for example, Faye et al. reported that meningitis-related hospitalization of children < 59 months remained stable despite the introduction of PCV-13 [46], but Mackenzie et al. reported that in Gambia, the introduction of PCVs reduced IPDs in children < 23 months by as much as 55% [47]. IPD rates also declined from the PCV-7 to PCV-13 era in the British Columbia of Canada [48]. Similar studies need to be conducted in Ghana to ascertain the gains made so far in the post-PCV-13 era.

Pneumococci were identified in eight of 114 (7.0%) culture positive cases of suppurative keratitis [20] as well as three of 89 (3.4%) of Gram-positive-related ear-discharge infections [11]. These reflect the relatively low frequency of pneumococci as aetiologic agents of noninvasive and nonmeningeal diseases compared to meningitis and hence the fewer studies in relation to other pneumococcal infections. In a study to determine the aetiology of acute lower respiratory tract infections among children under 5 years [49], pneumococci were found in one of eight blood (12.5%) samples, one of 22 throat swab (4.5%) samples, and three of 42 nasopharyngeal swab (7.1%) specimens presented for bacteriologic cultures, thus further suggesting their relatively low frequency as aetiologic agents of other diseases or their lower rate of successful isolation from these specimens. Studies involving other diseases may require greater sample sizes to obtain a significant number of isolates for a reportage or fall upon retrospective data to make any meaningful inference.

To assess the disease burden caused by the pneumococcus in Ghana, isolates from both invasive and non-IPDs should be considered to understand if there is any relationship between serotype and anatomical sites of infection as well as the dynamics associated with these.

Studies on pneumococcal diseases in Ghana have usually focused on meningitis, but a study of paediatric febrile illnesses conducted at the Agogo Presbyterian Hospital reported that pneumococci accounted for 134 of 209 (64%) of all cases of pneumonia [18]. In another study conducted at the Agogo Presbyterian Hospital, pneumococci were implicated in 9.1% (22/238) of all blood culture positives for children under 5 years hospitalized at the facility and were among the four most frequently isolated bacterial etiologic agents at a yearly cumulative incidence per 1000 being 4.3% [28]. In a laboratory-based nationwide surveillance study spanning a period of 6 months including 100 blood, 2 nasal, and 5 cerebrospinal fluid specimens, only two isolates of pneumococci were obtained [29]. The authors noted that most of the labs did not have the capacity to set up blood cultures. This may partly be a contributing factor to the lower reportage of other pneumococcal diseases aside from meningitis. Therefore, there may be an underestimation of the nonmeningeal IPDs in Ghana and, as such, a lower reported disease burden outside of the seasonal meningitis outbreaks.

### 4.4. Pneumococcal Serotypes Causing Disease

While there are over 100 distinct serotypes of pneumococci [42], only a few have been implicated in invasive infections [23]. Donkor et al., in their study on the population biology of the pneumococcus in West Africa, including isolates from Ghana, noted that serotypes commonly associated with invasive disease are seldom found in carriage [50]. This may be attributed to the fact that in Ghana, most cases of IPDs manifest as meningitis, primarily caused by Serotype 1, which, according to this study, appears to be rarely carried (Table 8). Although a few serotypes have been implicated in pneumococcal meningitis in Ghana, Serotype 1, a PCV13-included serotype, has been predominant [5]. Leimkugel et al. reported that 58/76 of pneumococcal meningitis in Kassena-Nankana District between 1998 and 2003 were all of Serotype 1 origin [23].

Apart from Serotype 1, other serotypes like 2, 3, 4, 8, 10F, 12F, 14, 35B, 38, and Serogroup 6 have also been isolated from meningitis cases (Table 3), but these are relatively rare in occurrence, are, for the most part (except 6A and 6B in Serogroup 6), non-PCV-13 serotypes, and are more commonly found in cases of carriage than in disease. However, Renner et al. have also specified that carriage is a prerequisite for infection [6]. Therefore, it is important to investigate whether the currently prevailing serotypes found in carriage are contributing significantly to pneumococcal diseases, both invasive and noninvasive, in the Ghanaian population.

Despite the introduction of PCV13, there was an increase in meningitis due to pneumococci Serotype 1 during 2016–2017 in the former Northern, Upper West, and Upper East regions [5]. Of 137 serotyped pneumococci in this outbreak [5], 20 (54%) were Serotype 1, followed by 2 of serotype 23F (5%), 2 of serotype 6A/6B (5%), and 2 of Serogroup 18 (5%) with Serogroups 3, 4, 5, 13, 19A, 19F, and 12 contributing to one case each. This was followed by Serotype 3 accounting for 7/76 cases, Serotypes 8 and 14 each accounting for 4/76 cases, Serotype 12 accounting for 3/76 cases, and other serotypes, that is, 2, 3, 4, 6A, 7F, 10F, and 38 each accounting for 1/76 cases [5]. Thus, four of the 11 implicated serotypes in the post-PCV-13 era were not PCV-13-incorporated serotypes. Of these, Serotype 8 was the most significant in terms of frequency of nonvaccine type-related meningitis [5].

Pneumococci accounted for 77% of positive cases of meningitis reported among children above 5 years of age in the Brong Ahafo Region, and 80% was due to Serotype 1 [22]. Furthermore, Bozio et al. found Serotype 1 to be a persistent cause of meningitis in the Northern and Upper regions of Ghana despite the introduction of PCV-13 into the national infant immunization programme [12]. This led to the study reporting that infant vaccination had not induced herd immunity in these regions. There is the need to conduct carriage studies in adults to find out if the adult population is not the cause of the persistence of this serotype in the population since Serotype 1 has been rarely isolated from carriage studies conducted among children.

### 4.5. Antibiotic Resistance

Denno et al. recommended the need for continuous surveillance to assess the prevalence of pneumococcus carriage, disease, and resistance patterns [17]. A national surveillance in Ghana reported that 11.8% of Gram-positive multidrug-resistant bacteria from blood stream infections were attributed to pneumococci, and these were recovered from children under 10 years [32]. This poses a major problem for the future treatment of pneumococcal diseases because the main drug of choice, Pen, is already under threat of resistance as previously reported [7].

The standard treatment for pneumococcal meningitis consists of Pen and Chl [20, 23]. For mild or moderate pneumonia, Amo or Pen were the drugs of choice [17]. Pen was also the chosen drug for the treatment of bacteremia without meningitis, but third generation cephalosporins were recommended by experts for the treatment of meningitis in areas of high prevalence of Pen-resistant pneumococci because Pen and Chl do not reach the required concentrations in the CSF [17]. There is the need to ascertain the efficacy of these antibiotics against the prevailing serotypes to obtain empiric data for purposes of treatment. There is also the need to ascertain whether the vaccine intervention has impacted positively against pneumococcal antibiotic resistance.

A study in Kassena-Nankana reported that 56 of 58 (96.6%) of Serotype 1 isolates from cases of meningitis were susceptible to Pen G, Ctx, Chl, and Cip [23], meaning resistance to some of the treatment options for pneumococcal diseases was underway in pre-PCV13 era. In the study of Dayie et al., pneumococci isolates were found to be intermediately resistant to Pen (37.4%), cotrimoxazole (85%), and Lev (2.5%), and the prevalence of multiple drug-resistant pneumococci was 34.3% [8]. Resistance patterns keep on changing; hence, it is possible for the nonvaccine serotypes to become highly resistant to current options for the treatment of pneumococcal diseases and thus nullify the current benefits of vaccine intervention. There is the need to emphasize prudent antibiotic usage and to assess new antibiotic resistance patterns at both the phenotypic and genotypic levels so that the relationship between resistance, serotypes, and sequence types (STs) can be elucidated. The time to consider changing the serotypes incorporated into PCVs for the region may soon be approaching if first-line drugs against the pathogen begin to fail.

### 4.6. Genetic Clonality of Pneumococcus

Capsular switching may be a contributing factor to the current disease burden of pneumococci in Ghana. There is still a potential threat of IPDs due to both the vaccine and nonvaccine serotypes in the postvaccine era, hence the need for continuous monitoring.

All Serotype 1 isolates from meningitis cases in Kassena-Nankana were found to be clonally related and showed 10 distinct STs [23] pointing to the fact that there may be a strong relation between pneumococci ST and IPD. Donkor et al., in studying the population biology of the pneumococcus in West Africa, stated that pneumococcal serotypes that are common in IPD were likely to be more clonal, for example, Serotypes 1 and 5, than the common ones found in carriage [50]. It has been reported that all ST of pneumococcus serotype 1 isolated from CSF in the Brong Ahafo region belonged to ST303 [22].

When sequencing the common serotype 19F isolated from both healthy children and those attending clinics in Ghana, it was found that nine out of the 14 identified STs were novel, and most of these were resistant to Pen [51]. From this population, the clones ST4190 and ST9090 were identified as clones specifically related to Ghana [51].

There is a paucity of data on the clonality of pneumococcus isolates from Ghana. Since pneumococci of the same serotype may differ genetically [51], there is the need for more sequencing studies to identify the clonality of both carriage and invasive pneumococci isolates as a means of monitoring the pathogenicity and antimicrobial resistance of the circulating serotypes. This is because serotypes that are presumed to be less invasive may potentially belong to clones associated with antimicrobial resistance and virulence. These may not be covered by current pneumococcal vaccines.

### 4.7. Implications for Future Research and Public Health

Primarily, continuous surveillance is needed to monitor changes in invasive disease serotypes as a means of evaluating the impact of PCV-13 especially on Serotype 1. Also, the 23-valent pneumococcal polysaccharide vaccine (PPV-23) should be administered to Ghanaian adults in regions near the meningitis belt during meningitis outbreak seasons as a means of reducing the disease burden associated with this serotype.

There is also the need for serotyping of all invasive disease and noninvasive disease isolates from hospitals as part of the pneumococcal surveillance to monitor changes due to serotype replacement. This may inform the need for future vaccine strategies.

In addition, there is the need to conduct carriage studies for other at-risk groups such as those living with chronic respiratory tract diseases since pneumococci are the main etiologic agents for bacterial pneumonia [52].

Next, continuous monitoring of pneumococcal antimicrobial-resistance patterns and detection of antimicrobial-resistance markers via genotyping of all disease isolates can provide a wealth of information needed to ensure that the current treatment guidelines for management of pneumococcal diseases in Ghana remain relevant. This is important because previous surveillance has shown that a range of clinical bacterial isolates in Ghana are resistant to important commonly used antimicrobials [53]. The natural competence of pneumococci means that these can easily acquire resistance genes horizontally from these resistant bacteria [54, 55].

Furthermore, there is the need to understand whether the persistence of the vaccine serotypes among children is due to the transmission of pneumococci from unvaccinated adult populations as well as the indirect impact of PCV-13 vaccination of infants on the adult population by conducting epidemiologic studies using unvaccinated adult populations.

Following on, 13 years after the introduction of PCV-13 into Ghana's Expanded Programme on Immunization, studies should be conducted among those who previously received PCV-13 vaccination during their infancy to ascertain their immunity to the PCV-13 pneumococcal vaccine serotypes.

Finally, the pneumococcal vaccine immune responsiveness of Ghanaian infants receiving PCV-13 should be determined as this has not been carried out in studies in Ghana since the introduction of the vaccine.

### 4.8. Limitations

To begin with, although the carriage studies [7–8, 15–17, 19, 25–26] considered the most vulnerable age groups in terms of the acquisition of IPDs, these are limited by the fact that those aged from 15 to 60 years are not exempted from IPD acquisition. This narrows the scope of the study because in epidemiological terms, the dynamics may be quite different from those of the other age groups considered.

Also, the clinical studies [10–13, 20–24, 28, 30–31] focused on a few health facilities within a few regions in Ghana, and geographic differences in terms of serotype distribution may occur between these regions. Studies from more health facilities in regions across the country could have played a significant role in ascertaining the disease burden due to the serotypes. Moreso, these studies were conducted at different times, and this could have influenced the carriage prevalence.

In addition, some studies [8, 12, 22, 25] did not report serotypes for some isolates but reported serogroups, and this may affect the overall prevalence of the different serotypes.

Furthermore, some of the studies [11, 13, 29, 30, 31] relied on retrospective data, and these are known to have an inferior level of evidence compared to other study designs.

Finally, fewer studies reported antimicrobial susceptibility results for agents such as rifampin [22] which, although not a first-line drug for the treatment of IPDs in Ghana, is important for future consideration as a combination treatment for meningitis in the rising trends of antimicrobial resistance.

## 5. Conclusion

This article has reviewed existing literature data on pneumococci in Ghana with the aim of gaining further insight into its disease burden, the relevance of prevailing serotypes, the menace of antibiotic resistance, and identifying research gaps regarding this pathogen. While it is known that carriage and serotype prevalence have been influenced by the introduction of PCV-13 in Ghana, the extent of this impact, nearly a decade later, remains unclear. There is a need to investigate whether the serotypes currently prevalent in carriage significantly contribute to the burden of pneumococcal diseases. Additionally, there have been fewer studies examining the role of pneumococci and their serotypes as etiological agents of other diseases, especially through blood cultures, as most studies have focused on nasopharyngeal carriage or cases of meningitis.

Also, predicting the invasiveness of pneumococci using molecular typing is the way to go in the future as this will provide answers to the extent to which capsular switching is contributing to the pneumococcal disease burden in Ghana almost a decade after introducing PCV-13. This is relevant because the nonvaccine serotypes possessing highly invasive genes of the vaccine serotypes may successfully evade the host immune system to cause diseases after establishing themselves as the predominant serotypes.

Furthermore, it is essential to determine whether vaccine interventions have had a positive impact on pneumococcal antibiotic resistance. Continuous monitoring of antibiotic resistance patterns at both phenotypic and genotypic levels, along with serotyping and molecular typing, should be standard practice in the surveillance of pneumococcal disease burden. This approach ensures public health safety and provides guidance for the empirical treatment of diseases caused by pneumococci.

## Figures and Tables

**Table 1:** Brief overview of methodologies used in reviewed studies.

**Study**	**Design**	**Variables**
Adjei and Agbemadzo [10]	Cross-sectional study	Antimicrobial susceptibility
Appiah-Korang et al. [11]	Retrospective study	Epidemiological data, clinical diagnosis, isolated organisms, and antimicrobial susceptibility.
Bozio et al. [12]	Cross-sectional study	Epidemiological data and serotype.
Dartey et al. [13]	Retrospective study	Epidemiological data and types of bacterial isolates.
Dayie et al. [7]	Cross-sectional study	Epidemiological data, serotype, and antimicrobial susceptibility.
Dayie et al. [14]	Cross-sectional study	Antimicrobial susceptibility and sequence types.
Dayie et al. [8]	Cross-sectional study	Epidemiological data, serotype, and antimicrobial susceptibility.
Dayie et al. [15]	Cross-sectional study	Epidemiological data and serotype.
Dayie et al. [16]	Cross-sectional study	Epidemiological data, serotype, antimicrobial susceptibility, and whole genome sequence.
Denno et al. [17]	Cross-sectional study	Epidemiological data and antimicrobial resistance.
Donkor, Foster-Nyarko, and Enweronu-Laryea [18]	Cross-sectional study	Epidemiological data and antimicrobial susceptibility.
Donkor et al. [19]	Cross-sectional study	Epidemiological data and serotype.
Hagan et al. [20]	Cross-sectional study	Species of bacterial agents.
Hogan et al. [21]	Prospective study	Epidemiological data, clinical diagnosis, and etiological agents.
Kwambana-Adams et al. [22]	Prospective study	Epidemiological data, serotypes, clinical diagnosis, antimicrobial susceptibility, and whole genome sequence types.
Leimkugel et al. [23]	Cross-sectional study	Epidemiological data, serotype, antimicrobial susceptibility, and sequence types.
Letsa et al. [24]	Case-control study	Epidemiological data, clinical diagnosis, and etiological agents.
Mills et al. [25]	Cross-sectional study	Epidemiological data, serotypes, and antimicrobial susceptibility.
Mills et al. [26]	Cross-sectional study	Epidemiological data, serotypes, antimicrobial susceptibility, and types of virulence genes.
Newman et al. [27]	Prospective study	Epidemiological data, antimicrobial resistance, and species of bacterial agents.
Nielsen et al. [28]	Cross-sectional study	Medical history, anthropometrical data, species of bacterial agents, and antimicrobial susceptibility.
Opintan and Newman [29]	Retrospective study	Antimicrobial susceptibility and species of bacterial agents.
Oteng et al. [30]	Retrospective study	Epidemiological data and species of bacterial agents.
Owusu et al. [31]	Retrospective study	Epidemiological data, species of bacterial agents, and antimicrobial agents.

**Table 2:** Carriage of *Streptococcus pneumoniae* in Ghana.

**Status**	**Prevalence of pneumococci (%)**	**Sample size**	**Age**	**Setting**	**Prevalence of penicillin-nonsusceptible pneumococci (%)**	**Reference**
Sick children	51.4	311	6–12 months	Komfo Anokye Teaching Hospital, Kumasi	17	Denno et al. [17]
Healthy children	54	410	< 5 years	Schools in Accra	22.3	Dayie et al. [16]
HIV patients	11	245	0.6–74 years	Korle Bu Teaching Hospital, Accra, and Princess Marie Louis Children's Hospital, Accra	28.6	Dayie et al. [15]
Sickle cell children	24.5	404	1–13 years and 14–82 years	Korle Bu Teaching Hospital, Accra, and Princess Marie Louis Children's Hospital, Accra	37.4	Dayie et al. [8]
Healthy children	33.3	848	< 6 years	Schools in Accra and Tamale	45.8	Dayie et al. [7]
HIV children	27.1	118	< 5–9 years and ≥ 9 years	Korle Bu Teaching Hospital, Accra	—	Donkor et al. [19]
Healthy children	29.4	513	≤ 24–59 months	Schools in Cape Coast	35	Mills et al. [26]
Children	48.9	423	< 5 years	Princess Marie Louis Children's Hospital, Accra	63	Mills et al. [25]

Abbreviation: HIV, human immunodeficiency virus.

**Table 3:** Distribution of PCV-13 carriage serotypes in Ghana.

**Serotype**	**Pre-PCV-13 era**	**Post-PCV-13 era**					**Total**
	**School carriers**	**Hospital carriers**	**HIV carriers**	**Sickle cell carriers**	**HIV carriers**	**KGs and centers**	
1	—	1 (0.1)	—	—	—	—	1 (0.1)
3	10 (1.1)	—	—	5 (0.5)	1 (0.1)	6 (0.7)	22 (2.4)
4	3 (0.3)	—	—	—	—	—	3 (0.3)
6A	21 (2.3)	—	3 (0.3)	2 (0.2)	—	6 (0.7)	32 (3.5)
6B	—	—	3 (0.3)	9 (1.0)	1 (0.1)	14 (1.5)	27 (2.9)
7C	—	—	2 (0.2)	—	—	2 (0.2)	4 (0.4)
9V	24 (2.6)	—	1 (0.1)	—	—	3 (0.3)	28 (3.0)
14	10 (1.1)	6 (0.7)	—	3 (0.3)	—	4 (0.4)	23 (2.5)
18C	4 (0.4)	—	—	—	—	1 (0.1)	5 (0.5)
19A	8 (0.8)	—	—	—	4 (0.4)	—	12 (1.3)
19F	37 (4.0)	—	10 (1.1)	—	1 (0.1)	11 (1.2)	59 (6.4)
23F	21 (2.3)	—	—	4 (0.4)	4 (0.4)	13 (1.4)	42 (4.6)
	Dayie et al. [7]	Mills et al. [25]	Donkor et al. [19]	Dayie et al. [8]	Dayie et al. [15]	Mills et al. [26]	258 (28.4)

Abbreviations: HIV, human immunodeficiency virus; KGs, kindergartens; Post-CV-13 era, 2012 to date; Pre-PCV-13 era, before 2012.

**Table 4:** Distribution of PCV-13 invasive serotypes in Ghana.

**Serotype**	**Pre-PCV-13 era**	**Post-PCV-13 era**		**Total**
	**Meningitis cases**	**Meningitis cases**	**Meningitis cases**	
1	63 (6.8)	38 (4.1)	85 (9.2)	186 (20.0)
3	7 (0.8)	1 (0.1)	1 (0.1)	9 (1.0)
4	1 (0.1)	—	1 (0.1)	2 (0.2)
5	—	—	4 (0.4)	4 (0.4)
6A	1 (0.1)	—	—	1 (0.1)
14	1 (0.1)	—	1 (0.1)	2 (0.2)
19A	—	—	1 (0.1)	1 (0.1)
19F	—	—	1 (0.1)	1 (0.1)
23F	—	—	3 (0.3)	3 (0.3)
	Leimkugel et al. [23]	Kwambana-Adams et al. [22]	Bozio et al. [12]	209 (22.4)

Abbreviations: PCV-13, 13-valent pneumococcal conjugate vaccine; Post-PCV-13 era, 2012 to date; Pre-PCV-13 era, before 2012.

**Table 5:** Distribution of PCV-13 invasive serotypes in Ghana.

**Serotype**	**School carriers**	**Hospital carriers**	**HIV carriers**	**Sickle cell carriers**	**HIV carriers**	**KGs and centers**	**Total**
6	—	26 (2.8)	—	—	—	—	26 (2.8)
6/10	—	1 (0.1)	—	—	—	—	1 (0.1)
7F	1 (0.1)	—	—	2 (0.2)	—	—	3 (0.3)
8	7 (0.8)	2 (0.2)	—	2 (0.2)	—	—	11 (1.2)
9A	—	—	—	1 (0.1)	—	—	1 (0.1)
9	—	1 (0.1)	—	—	—	—	1 (0.1)
9N	3 (0.3)	—	—	—	—	—	3 (0.3)
10	—	4 (0.4)	—	—	—	—	4 (0.4)
10A	—	—	1 (0.1)	5 (0.5)	2 (0.2)	5 (0.5)	13 (1.4)
11	—	5 (0.5)	—	—	—	—	5 (0.5)
11A	10 (1.1)	—	1 (0.1)	6 (0.7)	1 (0.1)	8 (0.9)	26 (2.8)
11D	—	—	—	1 (0.1)	—	—	1 (0.1)
12F	3 (0.3)	—	—	—	—	—	3 (0.3)
13	—	—	—	1 (0.1)	2 (0.2)	11 (1.2)	14 (1.5)
15	—	2 (0.2)	—	—	—	—	2 (0.2)
15A	—	—	—	3 (0.3)	1 (0.1)	6 (0.7)	9 (1.0)
15B	7 (0.8)	—	4 (0.4)	—	—	8 (0.9)	19 (2.1)
15C	—	—	3 (0.3)	4 (0.4)	—	1 (0.1)	8 (0.9)
16A	—	—	—	2 (0.2)	—	—	2 (0.2)
16F	—	—	9 (1.0)	—	2 (0.2)	1 (0.1)	12 (1.3)
17	—	2 (0.2)	—	—	—	4 (0.4)	6 (0.7)
17A	—	—	—	1 (0.1)	—	—	1 (0.1)
17F	1 (0.1)	—	—	1 (0.1)	1 (0.1)	—	3 (0.3)
18B	—	—	1 (0.1)	—	—	—	1 (0.1)
19B	—	—	—	1 (0.1)	1 (0.1)	6 (0.7)	8 (0.9)
19/6	—	1 (0.1)	—	—	—	—	1 (0.1)
19/14	—	1 (0.1)	—	—	—	—	1 (0.1)
19	—	17	—	5 (0.5)	—	—	22 (2.4)
20	2 (0.2)	1 (0.1)	—	—	—	1 (0.1)	4 (0.4)
21	—	—	—	1 (0.1)	—	5 (0.5)	6 (0.7)
21/29	—	1 (0.1)	—	—	—	—	1 (0.1)
23	—	7 (0.8)	—	—	—	—	7 (0.8)
23A	—	—	1 (0.1)	—	—	—	1 (0.1)
23B	—	—	2 (0.2)	7 (0.8)	1 (0.1)	22 (2.4)	32 (3.6)
24F	—	—	—	—	1 (0.1)	—	1 (0.1)
28F	—	—	—	1 (0.1)	1 (0.1)	—	2 (0.2)
31	—	—	—	1 (0.1)	—	1 (0.1)	2 (0.2)
32F	—	—	—	6 (0.7)	—	—	6 (0.7)
33	—	1 (0.1)	—	—	—	—	1 (0.1)
33A	—	—	—	—	1 (0.1)	—	1 (0.1)
33F	2 (0.2)	—	—	—	—	—	2 (0.2)
34	—	—	—	5 (0.5)	—	8 (0.9)	13 (1.4)
35B	—	—	—	—	—	2 (0.2)	2 (0.2)
38	—	—	—	1 (0.1)	—	1 (0.1)	2 (0.2)
Other NVT	59 (6.4)	—	—	—	—	—	59 (6.4)
NT	51 (5.5)	9 (1.0)	—	5 (0.5)	1 (0.1)	1 (0.1)	67 (7.2)
	Dayie et al. [7]	Mills et al. [25]	Donkor et al. [19]	Dayie et al. [8]	Dayie et al. [15]	Mills et al. [26]	416 (43.9)

Abbreviations: HIV, human immunodeficiency virus; KGs, kindergartens.

**Table 6:** Distribution of non-PCV-13 invasive serotypes in Ghana.

**Serotype**	**Pre-PCV-13 era**	**Post-PCV-13 era**		**Total**
	**Meningitis cases**	**Meningitis cases**	**Meningitis cases**	
6	—	1 (0.1)	1 (0.1)	2 (0.2)
7F	1 (0.1)	1 (0.1)	—	2 (0.2)
8	4 (0.4)	—	—	4 (0.4)
10F	1 (0.1)	—	—	1 (0.1)
12F	2 (0.2)	6 (0.7)	—	8 (0.9)
12/44/46	—	—	19 (2.1)	19 (2.1)
18	—	—	2 (0.2)	2 (0.2)
35B	—	2 (0.2)	—	2 (0.2)
38	1 (0.1)	—	—	1 (0.1)
	Leimkugel et al. [23]	Kwambana-Adams et al. [22]	Bozio et al. [12]	41 (4.4)

Abbreviations: PCV-13, 13-valent pneumococcal conjugate vaccine; Post-PCV-13 era, 2012 to date; Pre-PCV-13 era, before 2012.

**Table 7:** Antimicrobial resistance of pneumococci in Ghana.

**Setting**	**Population**	**Sample (number)**	**AST method**	**Prevalence of resistance (%)**	**Reference**
Wa Memorial Hospital in Navrongo or 1 of 4 health centers in the Kassena-Nankana District (1998–2003)	Patients < 1 yr to > 60 yrs with meningitis	CSF (76)	E-test	Pen G (0), Ctx (0), Chl (0), Cip (3.4)	Leimkugel et al. [23]
Nursery and kindergarten schools in Accra Metropolis and Tamale Metropolis (March–July, 2011)	848 children ≤ 6 yrs	Nasopharyngeal swabs (288)	Kirby-Bauer and E-test	Pen (45)	Dayie et al. [7]
KBTH (February 2006–April 2007)	92 sickle cell anemia patients < 12 yrs	Nasopharyngeal swabs (NA)	Kirby-Bauer	Pen (0), Amp (0), Clo (0), Crx (11), Ery (11), Cot (100)	Donkor, Foster-Nyarko, and Enweronu-Laryea [18]
Kindergartens and immunization centers in Cape Coast (February 2018)	513 children ≤ 24 to 59 months	NA	Kirby-Bauer and E-test	Pen (35), Lzd (0), Van (0), Lev (0), Ctr (2.6), Cli (5.3), Ery (7.3), Chl (11.3) MDR-28.5%	Mills et al. [26]
KBTH (February to April 2015)	118 HIV-infected children	Nasopharyngeal swabs (36)	Kirby-Bauer	Cot (58.3), Tet (33.3), Ery (33.3), Oxa (27.8), Ctr (5.6)	Donkor et al. [19]
KATH (NA)	NA	CSF (150)	E-test	Pen (31.3), Chl (20.6), Ery (12.6), Cot (18.0) MDR-14%	Adjei and Agbemadzo [10]
KATH Polyclinic and Immunization Clinic in Kumasi (June–August 1996)	311 children 6–12 mths	Nasopharyngeal (151)	Kirby-Bauer and E-test	Oxa (37.3), Tet (63.4), Chl (13.3), Ery (0), Ctx (0.7), Chl (8.7), Cot (47.8), Ctr (0), Crx (0)	Denno et al. [17]
2 teaching hospitals, 7 regional hospitals, and 2 district hospitals (December 2002–December 2003)	Patients	CSF, blood (51)	Kirby-Bauer and E-test	MDR-7.8%	Newman et al. [27]
APH (November 2013–April 2015)	1238 febrile paediatrics ≥ 30 days to ≤ 15 yrs	Blood (192)	Kirby-Bauer and E-test	Pen (76), Amo/Amp (77), Ctx (100), Ery/Azi (100), Cip (57), Gen (25), Tet (26), Chl (75)	Nielsen et al. [28]
KATH (1^st^ January 2008–31^st^ December 2010)	163 patients with meningitis	CSF (91)	Kirby-Bauer	Pen (1.1), Chl (17), Ctr (0), Ctx (0)	Owusu et al. [31]
Nursery and kindergarten schools in Accra Metropolis and Tamale Metropolis (NA)	848 children ≤ 6 yrs	Nasopharyngeal swabs (115)	Kirby-Bauer	Pen (90.4), Tmp (99.1), Tet (73), Smz (33.9), Ery (2.6), Ctx (5.2)	Dayie et al. [14]
Brong Ahafo Region (NA)	886 patients < 1 yrs to > 60 yrs with meningitis	CSF (NA)	Kirby-Bauer and E-test	Ctr (0), Van (0), Chl (0), Cli (0), Ery (0), Rif (0), Cot (76), Tet (80), Pen (11.7)	Kwambana-Adams et al. [22]
3 teaching hospitals, 7 regional hospitals, and 3 zonal public health reference laboratories (June–November 2014)	NA	NA (2)	Kirby-Bauer	Gen (16.7), Cip (50.0), Ery (66.7), Tet (83.3) MDR-100%	Opintan and Newman [29]
KBTH and PMLH (2016–2017)	402 sickle cell disease patients	Nasopharyngeal swabs (children: 79 and adults: 20)	Kirby-Bauer and E-test	Pen (37.4), Lev (2.5), Cot (85) MDR-34.3%	Dayie et al. [8]
Nurseries and kindergartens within Accra Metro (September–December 2016)	410 children < 5 yrs	Nasopharyngeal swabs (220)	Kirby-Bauer and E-test	Pen (22.3), Cot (61.4), Tet (63), Lev (0) MDR-20.5%	Dayie et al. [16]
KBTH and PMLCH (November 2016, March 2017)	245 HIV patients from < 5 to 80 yrs	Nasopharyngeal swabs (27)	Kirby-Bauer and E-test	Pen (11.1), Cot (92.6), Ery (7.4), Lev (0.0), Tet (66.7). MDR-18.5%	Dayie et al. [15]

Abbreviations: Amo, amoxicillin; Amp, ampicillin; APH, Agogo Presbyterian Hospital; AST, antimicrobial susceptibility test; Azi, azithromycin; Chl, chloramphenicol; Cip, ciprofloxacin; Clin, clindamycin; Clo, cloxacillin; Cot, cotrimoxazole; Crx, ceforoxime; Ctr, ceftriaxone; Ctx, cefotaxime; Ery, erythromycin; Gen, gentamicin; HIV, human immunodeficiency virus; KATH, Komfo Anokye Teaching Hospital; KBTH, Korle Bu Teaching Hospital; Lev, levofloxacin; Lzd, linezolid; MDR, multidrug resistance; mths, months; NA, not applicable; Oxa, oxacillin; Pen, penicillin; PMLCH, Princess Marie Louis Children's Hospital; Rif, rifampin; Smz, sulfamethoxazole; Tet, tetracyclin; Tmp, trimethoprim; Van, vancomycin; yrs, years.

**Table 8:** Diseases caused by *Streptococcus pneumoniae* in Ghana.

**Morbidity**	**Prevalence of *S. pneumoniae* (%)**	**Setting**	**Sample size**	**Age**	**Period**	**Reference**
Meningitis	19.2	Tamale	796	< 5 to 60 yrs	2016–2017	Bozio et al. [12]
Otitis media	3.4	Korle Bu Teaching Hospital, Accra	351	≤ 1 to 65+ yrs	2011–2013	Appiah-Korang et al. [11]
Pneumonia	64	—	299	≥ 30 days to 15+ yrs	2013–2015	Hogan et al. [21]
Meningitis	77.3	Jaman North, Brong Ahafo Region	44	0 to 50+ yrs	2016	Dartey et al. [13]
Meningitis	—	Komfo Anokye Teaching Hospital, Kumasi	150	—	—	Adjei and Agbemadzo [10]
Suppurative keratitis	4.0	Korle Bu Teaching Hospital, Accra	199	< 15 to 45+ yrs	—	Hagan et al. [20]
Meningitis	77	Brong Ahafo Region	135	< 1 to 60+ yrs	2015–2016	Kwambana-Adams et al. [22]
Meningitis	53.5	Kassena-Nankana	331	< 1 to 60+ yrs	1998–2003	Leimkugel et al. [23]
Meningitis	14.6	Brong Ahafo Region	969	> 5 to 50+ yrs	2015–2016	Letsa et al. [24]
Meningitis	77.7	Komfo Anokye Teaching Hospital, Kumasi	163	≤ 1 mth to 50+ yrs	2008–2010	Owusu et al. [31]
Bacteremia	9.1	Agogo Presbyterian Hospital	238	< 5 yrs	2007–2009	Nielsen et al. [28]
Meningitis	100	St. Joseph's Hospital, Jirapa	10	< 10 to 69+ yrs	2017	Oteng et al. [30]

Abbreviations: mth, month; yrs, years.

## Data Availability

The authors have nothing to report.
